# AMPA Receptor Trafficking for Postsynaptic Potentiation

**DOI:** 10.3389/fncel.2018.00361

**Published:** 2018-10-11

**Authors:** Mikyoung Park

**Affiliations:** ^1^Center for Functional Connectomics, Brain Science Institute, Korea Institute of Science and Technology (KIST), Seoul, South Korea; ^2^Department of Neuroscience, Korea University of Science and Technology, Daejeon, South Korea

**Keywords:** AMPA receptors, long-term potentiation, postsynapse, exocytosis, lateral diffusion

## Abstract

Long-term potentiation (LTP) of excitatory synaptic strength, which has long been considered a synaptic correlate for learning and memory, requires a fast recruitment of additional α-amino-3-hydroxy-5-methyl-4-isoxazolepropionate (AMPA) receptors (AMPARs) to the postsynaptic sites. As cell biological concepts have been applied to the field and genetic manipulation and microscopic imaging technologies have been advanced, visualization of the trafficking of AMPARs to synapses for LTP has been investigated intensively over the last decade. Recycling endosomes have been reported as intracellular storage organelles to supply AMPARs for LTP through the endocytic recycling pathway. In addition, exocytic domains in the spine plasma membrane, where AMPARs are inserted from the intracellular compartment, and nanodomains, where diffusing AMPARs are trapped and immobilized inside synapses for LTP, have been described. Furthermore, cell surface lateral diffusion of AMPARs from extrasynaptic to synaptic sites has been reported as a key step for AMPAR location to the synaptic sites for LTP. This review article will discuss recent findings and views on the reservoir(s) of AMPARs and their trafficking for LTP expression by focusing on the exocytosis and lateral diffusion of AMPARs, and provide some future directions that need to be addressed in the field of LTP.

## Introduction

Synapses are fundamental units of brain function and possess the remarkable ability to change their strength in function and structure through synaptic plasticity. Long-term potentiation (LTP), a well characterized form of synaptic plasticity that has long been considered a synaptic correlate for learning and memory, was discovered in the hippocampus in 1973 (Bliss and Lømo, 1973). Prior to the discovery of LTP, Hebb’s (1949) postulate that learning and memory involves synaptic strengthening elicited by the coordinated firing of pre- and postsynaptic cells was suggested. In addition, beginning with Ramón y Cajal (1852–1934), many neuroscientists have suggested that learning and memory should involve synaptic modifications (Malenka, 2003). A type of glutamate receptor, the α-amino-3-hydroxy-5-methyl-4-isoxazolepropionate (AMPA) receptor (AMPAR), has been investigated intensively as a key player in synaptic modifications involved in synaptic transmission, synaptic plasticity and, ultimately, learning and memory. LTP and long-term depression (LTD), another well characterized form of synaptic plasticity in the hippocampus, are expressed by long lasting changes of AMPAR-mediated synaptic responses. Exocytosis and endocytosis of AMPARs play critical roles in LTP and LTD, respectively, in aspects of both functional and structural plasticity of synapses (Kessels and Malinow, 2009; Anggono and Huganir, 2012; Huganir and Nicoll, 2013). Indeed, learning induces LTP in the hippocampus (Whitlock et al., 2006). Learning alters AMPAR phosphorylation and synaptic delivery of AMPARs (Whitlock et al., 2006), which are readouts for LTP (Heynen et al., 2000; Lee et al., 2000; Malenka, 2003). Although studies of LTP were conducted originally through electrophysiological approaches, advances in the tools of molecular and cellular biology, biochemistry, state-of-the-art imaging and genetics have provided much more sophisticated information of AMPAR trafficking to synapses to support LTP mechanisms. This review article provides a brief introduction of AMPARs and LTP, followed by a focus on recent findings and views on AMPAR reservoir(s) for LTP by examining studies on the exocytosis and cell surface lateral diffusion of AMPARs during LTP.

## AMPARs and LTP

AMPARs are major ionotropic glutamate receptors that respond to physiological glutamate, a major excitatory neurotransmitter in the mammalian central nervous system. AMPARs have four subunits, GluA1–GluA4 encoded by *Gria1*–*Gria4* genes, and those subunits form hetero-tetramers composed of two dimers (Wisden and Seeburg, 1993; Hollmann and Heinemann, 1994; Dingledine et al., 1999; Traynelis et al., 2010; Chater and Goda, 2014). The combination of each subunit forms a developmentally distinct receptor complex in the hippocampus (Wenthold et al., 1996; Zhu et al., 2000). Immature hippocampal neurons at early developmental stages express the GluA4 subunit, which complexes with the GluA2 subunit (Zhu et al., 2000). However, mature hippocampal neurons express two predominant combinations of AMPAR subunits, GluA1/GluA2 or GluA2/GluA3 heterotetrameric receptors (Wenthold et al., 1996). Regulation of the precise localization and number of AMPARs at the cell surface membrane is critical for most excitatory synaptic transmission at the steady state and also for long-term synaptic plasticity, such as LTP and LTD (Song and Huganir, 2002; Bredt and Nicoll, 2003).

The majority of studies on LTP have been performed on excitatory synapses between Schaffer collateral-commissural axons and CA1 pyramidal neuron dendrites in the hippocampus (Bear and Kirkwood, 1993; Kirkwood et al., 1993; Nicoll and Roche, 2013). While LTP is triggered rapidly by a brief high-frequency stimulation (HFS), it persists for days or even weeks *in vivo*. The “early phase” of LTP, which lasts approximately 60 min, requires the activation of *N-methyl*-D-aspartate (NMDA) receptors (NMDARs) for its induction, together with subsequent Ca^2+^ influx and calcium/calmodulin-dependent protein kinase II (CaMKII) activation (Malenka et al., 1989; Malinow et al., 1989; Silva et al., 1992; Lisman, 1994; Lisman et al., 1997; Malenka and Nicoll, 1999). In addition, delivery of new AMPARs to the postsynaptic sites is believed to be responsible for LTP expression in its early phases. One distinguishable characteristic of the “late phase” of LTP, which lasts days or even weeks, from the “early phase” of LTP, is that the late phase requires gene transcription and new protein synthesis (Schuman et al., 2006; Reymann and Frey, 2007; Johnstone and Raymond, 2011; but also see Abbas et al., 2009; Villers et al., 2012).

Three major questions have been the focus of studies in the field of LTP. First, studies examined whether the increase in synaptic strength during LTP at Schaffer collateral-CA1 synapses is due primarily to presynaptic or postsynaptic modifications (Kullmann and Siegelbaum, 1995; Nicoll and Malenka, 1995; Emptage et al., 1999, 2003; Malenka and Nicoll, 1999; Ward et al., 2006; Kerchner and Nicoll, 2008; Enoki et al., 2009; Kullmann, 2012; Chater and Goda, 2014; Granger and Nicoll, 2014; Padamsey and Emptage, 2014). Second, studies have been conducted to determine which AMPAR subunits are responsible for LTP expression (Jia et al., 1996; Zamanillo et al., 1999; Hayashi et al., 2000; Shi et al., 2001; Granger et al., 2013; Granger and Nicoll, 2014; Diaz-Alonso et al., 2017; Zhou et al., 2018). Finally, studies have examined whether AMPARs are located to the synapse for LTP expression through exocytosis and/or lateral diffusion. The debate about whether the location of LTP expression at CA1 synapses is presynaptic or postsynaptic has lasted for more than two decades. Currently, most data, even those previously supporting a presynaptic change of increased release probability or decreased synaptic failure in LTP expression can be reconciled with postsynaptic changes by the “silent synapse” concept (Isaac et al., 1995, 1996; Liao et al., 1995, 1999; Durand et al., 1996; Gomperts et al., 1998; Nusser et al., 1998; Petralia et al., 1999; Takumi et al., 1999; Kerchner and Nicoll, 2008). It is now generally accepted that LTP expression at Schaffer collateral-CA1 synapses is mediated by AMPAR insertion into the synapse, supporting the postsynaptic view for LTP expression (Malenka and Nicoll, 1999; Shi et al., 1999; Hayashi et al., 2000; Malinow and Malenka, 2002; Song and Huganir, 2002; Bredt and Nicoll, 2003; Nicoll, 2003; Chater and Goda, 2014; Granger and Nicoll, 2014). Single channel conductance increases of AMPARs have been suggested to mediate LTP expression in the CA1 region of the hippocampus (Benke et al., 1998). However, a recent reevaluation of this study by the same group showed that insertion of AMPARs with high conductance can account for LTP expression (Benke and Traynelis, 2018). In addition to postsynaptically expressed LTP at Schaffer collateral-CA1 synapses, a distinct form of LTP at mossy fiber synapses, which is independent of NMDARs and expressed presynaptically unlike that at Schaffer collateral-CA1 synapses, has been well investigated and extensivley discussed (Nicoll and Malenka, 1995; Nicoll and Schmitz, 2005; Granger and Nicoll, 2014). AMPARs, particularly those containing the GluA1 subunit, have been suggested to play an important role in LTP expression at CA1 synapses in studies using knockout mice lacking GluA1 or GluA2 and electrophysiological recordings of hippocampal slice expressing tagged GluA1 or GluA2 (Jia et al., 1996; Zamanillo et al., 1999; Hayashi et al., 2000; Shi et al., 2001; Diaz-Alonso et al., 2017). The GluA1 subunit requirement for LTP has been investigated and supported by studies focusing on the cytoplasmic carboxy terminal (C-terminal) tail, which has been demonstrated to be involved in intracellular signaling through phosphorylation, palmitoylation or protein interactions (Barria et al., 1997; Hayashi et al., 2000; Shi et al., 2001; Esteban et al., 2003; Lee et al., 2003; Boehm et al., 2006; Lin et al., 2009). However, the GluA1 C-terminal tail requirement for LTP was challenged by a report showing that LTP requires AMPAR trafficking, independent of subunit type (Granger et al., 2013). Interestingly, recent studies have demonstrated that the extracellular amino-terminal domain (ATD) of AMPARs governs their trafficking for synaptic plasticity dependent on the AMPAR subunit type (Diaz-Alonso et al., 2017; Watson et al., 2017). Further, the spatial resolution of AMPARs delivered into the synapse for LTP has been questioned whether it is through exocytosis directly from the intracellular pool to synaptic sites or through lateral mobility from the extrasynaptic plasma membrane or a combination of each (Lledo et al., 1998; Lu et al., 2001; Park et al., 2004; Kopec et al., 2006, 2007b; Yudowski et al., 2007; Jaskolski and Henley, 2009; Lin et al., 2009; Makino and Malinow, 2009; Petrini et al., 2009; Kennedy et al., 2010; Opazo et al., 2010; Patterson et al., 2010; Cho et al., 2015; Penn et al., 2017; Temkin et al., 2017; Wu et al., 2017).

## Exocytosis and Lateral Mobility of AMPARs for LTP

Much evidence suggests that LTP expression is mediated by postsynaptic mechanisms (Lisman et al., 2012; Lu and Roche, 2012; Granger et al., 2013; Granger and Nicoll, 2014) and requires exocytosis at or near the postsynaptic membrane, which results in an increase in the number of AMPARs (Lledo et al., 1998; Lu et al., 2001; Park et al., 2004; Patterson et al., 2010; Ehlers, 2013; Wu et al., 2017). Initial studies showed that introducing various reagents that disrupt membrane fusion into the postsynaptic cells blocks LTP at Schaffer collateral-CA1 synapses (Lledo et al., 1998), implying that the exocytosis of intracellular vesicles harboring AMPARs is an essential step for LTP. A serial electron microscopy study demonstrated that endosomal compartments are located in dendrites and dendritic spines and serve as intracellular storehouses for the plasma membrane (Cooney et al., 2002). Some molecules that are retained on endosomal compartments can be delivered rapidly to the cell surface in response to stimuli (Lampson et al., 2001; Bryant et al., 2002; Cooney et al., 2002; Zeigerer et al., 2002; Govers et al., 2004; Guilherme et al., 2004). In addition, another study showed that synaptic activity that can induce LTP drives AMPARs to be endocytosed and reinserted to the plasma membrane (Ehlers, 2000). Together, these data suggest that endosomal organelles involved in endocytic recycling transport can serve as primary intracellular membrane compartments mobilized to the plasma membrane in response to LTP-inducing stimuli (Ehlers, 2013). Indeed, disrupting the transport of recycling endosomes to the plasma membrane using dominant negative forms of Rab11 (Ullrich et al., 1996; Zerial and Mcbride, 2001), syntaxin 13 (Prekeris et al., 1998), or Eps15 homology domain protein Rme1/EHD1 (Grant et al., 2001; Lin et al., 2001) blocked synaptic delivery of AMPARs during LTP (Park et al., 2004). Postsynaptic synaptotagmin-1 and synaptotagmin-7 were reported to mediate GluA1 exocytosis during LTP by acting as postsynaptic Ca^2+^-sensors (Wu et al., 2017). Complexin, a regulator of SNARE-mediated neurotransmitter release in presynapses (Reim et al., 2001; Maximov et al., 2009), binds to SNARE complexes to mediate AMPAR exocytosis during LTP in postsynapses (Ahmad et al., 2012). Subsequently, postsynaptic SNARE proteins such as syntaxin 3, SNAP-47 and synaptobrevin-2, which are distinct from proteins involved in presynaptic neurotransmitter release, were reported to regulate AMPAR exocytosis during LTP (Jurado et al., 2013).

Visualization of activity-triggered exocytosis of AMPARs in dendrites and dendritic spines is possible using the pH-sensitive superecliptic pHluorin (SEP), whose fluorescence is quenched at low pH (Miesenbock et al., 1998). Indeed, using SEP-tagged AMPARs, the postsynaptic exocytosis of AMPARs during LTP has been visualized directly (Kopec et al., 2006, 2007a; Yudowski et al., 2007; Lin et al., 2009; Makino and Malinow, 2009; Petrini et al., 2009; Araki et al., 2010; Kennedy et al., 2010; Patterson et al., 2010; Cho et al., 2015). Glycine-induced LTP has been shown to be mediated by an accumulation and immobilization of SEP-GluA1s at synapses, due to both exocytosis and stabilization of GluA1s at the postsynaptic density (PSD; Petrini et al., 2009). Impairment of GluA1 recycling exocytosis with a dominant-negative mutant of Rab11 results in GluA1 being less mobile at synapses. In addition, the displacement of endocytic zones from the PSD by a point mutant of dynamin-3 unable to bind Homer1 (Lu et al., 2007) impairs glycine-induced LTP expression by blocking GluA1 recycling (Petrini et al., 2009). Taken together, these results suggest that the GluA1 endocytic recycling pool is crucial for maintaining a mobile population of surface GluA1s that can be mobilized to synapses for LTP. Bath application of glycine for inducing LTP increases the exocytic events of SEP-GluA1 in dendrites and dendritic spines (Yudowski et al., 2007; Cho et al., 2015). In a more localized activation using two-photon glutamate uncaging, which mimics single synaptic release with sufficient spatiotemporal resolution (Matsuzaki et al., 2004; Bagal et al., 2005; Harvey and Svoboda, 2007; Lee et al., 2009), SEP-GluA1 was observed to be exocytosed to dendrites and activated spines (Makino and Malinow, 2009; Patterson et al., 2010). The increase of AMPAR-mediated currents was observed in spines initially, and then in the dendrite following glutamate uncaging-evoked LTP (Makino and Malinow, 2009), consistent with GluA1 insertion directly to the spines.

Many studies have supported the idea that LTP triggers the exocytosis of AMPARs required for expression. In addition, several studies have demonstrated how the intracellular recycling endosome that stores AMPARs is mobilized to near or at the spine for LTP, and where AMPARs are exocytosed to the spine surface in relation to the PSD for LTP (Wang et al., 2008; Kennedy et al., 2010). The actin-based Ca^2+^-sensitive motor protein myosin Vb has been reported to mediate the translocation of recycling endosomes harboring AMPARs into spines during LTP (Wang et al., 2008). Blockade of myosin Vb using RNA interference or chemical-genetic inhibition results in reduced LTP-induced SEP-GluA1 insertion and hippocampal slice LTP, indicating that the myosin Vb-mediated mobilization of recycling endosomes is required for synaptic potentiation (Wang et al., 2008). Related actin-based myosin Va has also been reported to mediate the translocation of AMPARs to spines from the dendritic shaft during LTP (Correia et al., 2008). Neurons expressing a dominant-negative form of myosin Va or a short interfering RNA specific for myosin Va showed a blockade of synaptic delivery of GluA1 and LTP (Correia et al., 2008). Conversely, myosin Va mutant mice showed normal synaptic plasticity (Schnell and Nicoll, 2001), suggesting a potential compensation by other Class V myosins. In a recent report on another actin-dependent motor protein myosin IXa, myosin IXa^+/–^ mice displayed impaired LTP (Folci et al., 2016), together indicating that myosin motor proteins play roles in AMPAR delivery during LTP. Using SEP-GluA1 and transferrin receptor (TfR), a classic recycling endosomal marker, GluA1 in TfR-positive recycling endosomes was shown to be exocytosed to spines adjacent to the PSD during glycine-induced LTP. Newly inserted SEP-GluA1 either quickly diffuses out of the spine or stays near the site of fusion in spines, whereas TfRs that co-exocytosed with SEP-GluA1 always diffused out of the spine immediately following the co-appearance of SEP-GluA1 (Kennedy et al., 2010). The exocytic events occurring adjacent to the PSD are mediated by syntaxin-4, which played a role in recycling endosome fusion to the spine plasma membrane. Disrupting syntaxin-4 blocks spine exocytosis and impairs LTP (Kennedy et al., 2010). The results of this study also suggest that different cargoes follow their own fate once they arrive at the spine surface. Although this study reported a requirement for syntaxin-4, but not for syntaxin-3 in LTP (Kennedy et al., 2010), other groups have demonstrated that LTP requires syntaxin-3, but not syntaxin-4 (Jurado et al., 2013; Arendt et al., 2015). Several explanations for these contradictory results have been extensively debated in the “Discussion” section of Jurado et al. (2013).

Recent work from Choquet and co-workers has provided a temporal profile of AMPAR trafficking for LTP expression by employing a novel approach that immobilizes surface AMPARs to prevent their diffusion on the cell surface (Penn et al., 2017). Biotin-tethered AMPAR subunit GluA1 or GluA2 can be expressed exogenously in cultured hippocampal neurons along with the endoplasmic-reticulum-retained biotin ligase (BirA-ER). In the presence of the biotin-binding protein NeutrAvidin, biotin-tethered AMPARs can be effectively crosslinked by NeutrAvidin, which reduces their surface diffusion as monitored by fluorescence recovery after photobleaching (FRAP; Penn et al., 2017). Using biotin-tethered GluA2 exogenously expressed in organotypic hippocampal slices prepared from GluA2-knockout mice, it was demonstrated that acute pre-treatment with NeutrAvidin to immobilize only pre-existing surface GluA2 results in a complete blockade of the short-term potentiation induced by a HFS LTP protocol. However, the hippocampal cells still express a detectable LTP, although small, indicating a contribution of exocytosis for LTP expression. Accordingly, the prevention of postsynaptic membrane fusion events by the intracellular application of tetanus toxin blocked HFS-induced LTP completely, but normal levels of short-term potentiation were still expressed, indicating a requirement of exocytosis for LTP expression (Penn et al., 2017).

**Figure 1:**
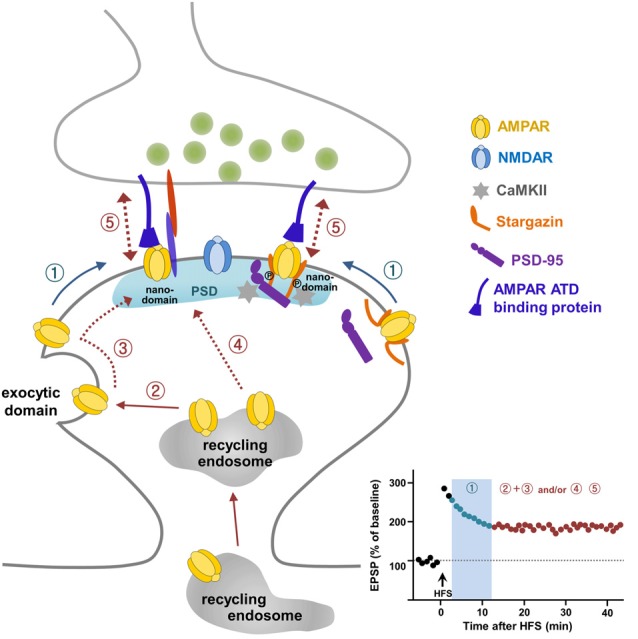
A current model for α-amino-3-hydroxy-5-methyl-4-isoxazolepropionate (AMPA) receptor (AMPAR) trafficking during long-term potentiation (LTP). Pre-existing extrasynaptic surface AMPARs are mobilized to the synaptic sites upon LTP stimulation via lateral diffusion (①) and are trapped in nanodomains within the synapse, which is required for short-term potentiation (① in LTP graph). AMPARs exocytosed from intracellular recycling endosomes to the exocytic domain adjacent to the postsynaptic density (PSD) replenish the extrasynaptic surface pool of AMPARs (②), and these exocytosed AMPARs are mobilized laterally to reach the synaptic site (③) and supply AMPARs for maintaining LTP expression. The direct delivery of a portion of AMPARs on recycling endosomes to the synaptic site during LTP (④) cannot be ruled out. AMPARs trapped at the synapse are immobilized through the regulation of Stargazin and probably through the subunit-specific regulation of the AMPAR amino-terminal domain (ATD) interacting protein (⑤).

It is very clear that LTP requires AMPAR exocytosis to sites adjacent to the PSD. These exocytosed receptors need to be relocated to synapses for synaptic potentiation (Makino and Malinow, 2009; Kennedy et al., 2010; Patterson et al., 2010). Interestingly, some populations of AMPARs may diffuse in and out between extrasynaptic and synaptic sites in order to tune synaptic transmission (Heine et al., 2008), and this receptor exchange organized on the surface membrane through lateral mobility is regulated dynamically by activity. Choquet and co-workers demonstrated that extrasynaptic surface AMPARs adjacent to the PSD arrive at synaptic sites through lateral diffusion upon LTP stimulation (Tardin et al., 2003; Opazo et al., 2010; Opazo and Choquet, 2011; Huganir and Nicoll, 2013; Chater and Goda, 2014; Constals et al., 2015; Compans et al., 2016; Penn et al., 2017). The lateral mobility of the surface GluA2 subunit inside nanodomains and/or outside of synapses was observed first using single-molecule fluorescence microscopy (Borgdorff and Choquet, 2002; Tardin et al., 2003). Lateral diffusion of surface GluA2 between extrasynaptic and synaptic sites is regulated by increased intracellular Ca^2+^, glutamate application, and glycine-induced stimulation, suggesting that the lateral diffusion of AMPARs may act as an important controlling step for synaptic plasticity (Borgdorff and Choquet, 2002; Tardin et al., 2003). Taken together, these reports suggest that a pre-existing surface pool of AMPARs are a prompt source to reach synapses via lateral diffusion and are then trapped at synapses for short-term potentiation, while newly exocytosed AMPARs from the recycling endosome upon LTP stimulation are the major source for sustaining LTP expression (Figure 1). Future work should address the intriguing possibility that the prompt reservoir of AMPARs for LTP is an extra-nanodomain or a true extrasynaptic region.

## Stabilization of AMPARs at Synaptic Sites for LTP

Laterally diffusing surface AMPARs must be trapped and immobilized at nanodomains on synaptic sites for LTP stabilization. Stargazin, an AMPAR auxiliary protein (Tomita et al., 2005; Hafner et al., 2015), was reported as a key molecule involved in the trapping and stabilization of AMPARs at synaptic sites during LTP (Opazo et al., 2010). LTP-inducing stimulation to activate NMDARs resulting in Ca^2+^ influx triggers CaMKII activation. CaMKII-mediated phosphorylation of the C-terminal PDZ-binding domain in Stargazin creates a highly negatively charged C-terminal tail of Stargazin so that it repulses the negatively charged membrane lipid. The C-terminal tail of Stargazin then unfolds, which favors its binding to PSD-95, thereby increasing the synaptic trapping of AMPARs on the nanodomains (Figure 1; Opazo et al., 2010; Opazo and Choquet, 2011; Choquet and Triller, 2013).

Besides these intracellular mechanisms of AMPAR trapping and stabilization at synaptic sites, LTP stabilization might also involve trans-synaptic mechanisms involving the extracellular ATD of AMPARs. Indeed, the involvement of extracellular domains of AMPARs in LTP has recently been reported (Diaz-Alonso et al., 2017; Watson et al., 2017). The extracellular ATDs of GluA1 and GluA2 exert a subunit-specific role in synaptic trafficking of AMPARs. The ATD of GluA1, but not GluA2, is required for surface GluA1 translocation to synapses (Diaz-Alonso et al., 2017). GluA1 without ATD exhibits increased mobility in synapses and failed to sustain LTP (Diaz-Alonso et al., 2017), indicating a requirement for ATD of GluA1 in LTP stabilization. Further, a concept of a trans-synaptic molecular nanocolumn, stretching from the presynaptic neurotransmitter release site to the postsynaptic receptor cluster, has been introduced (Savtchenko and Rusakov, 2014; Tang et al., 2016; Biederer et al., 2017). LTD-triggering stimuli reorganized nanocolumns through trans-synaptic nanocluster realignment whereas LTP-triggering stimuli reorganized only postsynaptic nanoclusters with no changes in presynaptic nanoclusters (Tang et al., 2016). It might be possible to observe presynaptic nanocluster reorganization if LTP could be stabilized, which can be mediated by trans-synaptic communications.

Super-resolution imaging technologies (Hell and Wichmann, 1994; Betzig, 1995; Betzig et al., 2006; Rust et al., 2006; Manley et al., 2008) with 10- to 100-nm spatial resolution, such as stimulated emission depletion microscopy (STED), photoactivated localization microscopy (PALM), universal point accumulation in nanoscale topography (u-PAINT), direct stochastic optical reconstruction microscopy (dSTORM) and electron microscopy have demonstrated AMPAR nanodomains inside synapses, with 1–3 of 80 nm clusters at each synapse and 20–25 AMPARs in each cluster (Nair et al., 2013), and trans-synaptic nanocolumns (Tang et al., 2016). Improvements in super-resolution imaging techniques and protein sensor development should allow greater manipulation and observation (Chater and Goda, 2014; Granger and Nicoll, 2014; Martineau et al., 2017). These improved methods will facilitate investigations as to whether new nanodomains, where AMPARs are trapped during LTP, and/or nanocoulmns are formed to mediate synaptic potentiation during LTP and whether AMPARs are trapped on pre-existing and/or newly formed nanodomains and/or nanocolumn, if generated, during LTP (Compans et al., 2016).

## Summary and Perspectives

Over the past two decades, many laboratories have committed intensive effort to uncover mechanisms underlying AMPAR trafficking during LTP. These efforts, employing novel and advanced methods in electrophysiology, molecular and cellular biology, biochemistry, imaging and genetics provide a working model for how reserve pools of AMPARs are delivered to synapses for LTP (Figure 1). According to this model, pre-existing surface AMPARs are the first requirement for LTP expression. These pre-existing surface AMPARs are mobilized quickly to synaptic sites via lateral diffusion upon LTP stimulation and are trapped in microdomains within synapses (Figure 1①). To sustain the expression of LTP, more AMPARs need to be delivered to the synaptic sites. This occurs via exocytosis of AMPARs from the recycling endosome to extrasynaptic sites to replenish the surface pool of AMPARs (Figure 1②), which then diffuse laterally and are trapped at the synapses (Figure 1③). In addition, it cannot be ruled out that a portion of AMPARs are supplied to the synaptic sites via exocytosis directly from the recycling endosomes (Figure 1④). Although the study by Penn et al. (2017) has advanced our understanding of LTP expression, some questions still remain to be addressed. Future development of molecular and opto-genetic manipulations and imaging technologies with greater spatial and temporal resoultion, will help determine whether subunits of AMPARs interplay with each other to contribute differentially to LTP expression. In addition, it will be of interest to investigate the relationship between AMPAR surface mobility and LTD, and, further, to elucidate whether different learning paradigms such as fear conditioning, water maze, passive avoidance, or novel object recognition utilize specific AMPAR trafficking mechanisms. Indeed, the C-terminal tails of GluA1 and GluA2 have been reported to exert differential roles in spatial learning and memory and contextual fear memory, respectively, suggesting a specific regulation of behavioral plasticity by AMPARs (Zhou et al., 2018).

The requirement of the C-terminal tail of GluA1 for LTP has been well accepted in the field since the sophisticated electrophysiology study of Shi et al. (2001). This work has resulted in many follow-up studies, whose main focus has been uncovering the nature of the molecules interacting with the C-terminal tails of AMPARs for the regulation of synaptic transmission and plasticity. However, the requirement of the C-terminal tail of GluA1 for LTP has been challenged (Granger et al., 2013), and subsequent studies have turned their attention toward the involvement of extracellular domains of AMPARs in LTP (Diaz-Alonso et al., 2017; Watson et al., 2017). Mobilization of surface AMPARs from extrasynaptic to synaptic sites is a well-recognized process for LTP expression. Stargazin has been identified as a regulator for LTP-triggered CaMKII-mediated trapping and immobilization of AMPARs diffusing in the membrane (Opazo et al., 2010). Analogous to the intracellular mechanism underlying immobilization of AMPARs at synapses by Stargazin, and given that studies highlight the importance of extracellular ATDs of AMPARs in LTP (Diaz-Alonso et al., 2017; Watson et al., 2017) and the reorganization of trans-synaptic nanocolumns by NMDAR activation (Tang et al., 2016), it is possible that trans-synaptic anchoring mechanisms, probably involving synaptic adhesion molecules, stabilize AMPARs by trapping them through their ATDs and preventing them from diffusing during LTP (Figure 1⑤). Some synaptic adhesion molecules may be good candidates for anchoring through direct or indirect interactions with the ATD of AMPARs in the cleft space (Shipman and Nicoll, 2012; Aoto et al., 2013; Anderson et al., 2015; Jang et al., 2016; Gulisano et al., 2017; Varbanov and Dityatev, 2017; Bhouri et al., 2018). Further studies of extracellular trans-synaptic ATD regulation will add more information about the specific mechanisms by identifying the molecules involved in AMPAR trafficking during LTP. The recent visualization and measurement of intracellular transport of newly synthesized AMPARs during LTP (Hangen et al., 2018) provides a possible way to investigate the intracellular dynamics and mechanisms of synaptic key molecules that link the transition from the early to late phase of LTP, which might be contributed by local protein synthesis in dendrites (Sutton and Schuman, 2005; Sutton et al., 2006). Together, these approaches will further expand our understanding of LTP and open a new era in studies into how LTP affects synaptic plasticity and ultimately learning and memory.

## Author Contributions

The author confirms being the sole contributor of this work and approved it for publication.

## Conflict of Interest Statement

The author declares that the research was conducted in the absence of any commercial or financial relationships that could be construed as a potential conflict of interest.
